# Biomaterial Techniques for Enhancing CAR-T Cell Therapy of Solid Tumours

**DOI:** 10.3390/cancers18172727

**Published:** 2026-08-22

**Authors:** Kai Chilvers, John Maher

**Affiliations:** 1 School of Medicine, King’s College London, 1st Floor, Henriette Raphael Building, Guy’s Hospital Campus Great Maze Pond, London SE1 1UL, UK; kai.chilvers@kcl.ac.uk; 2 CAR Mechanics Group, Guy’s Cancer Centre, School of Cancer and Pharmaceutical Sciences, King’s College London, Great Maze Pond, London SE1 9RT, UK; 3 Leucid Bio Ltd., Guy’s Hospital, Great Maze Pond, London SE1 9RT, UK; 4 Department of Immunology, Eastbourne Hospital, Kings Drive, Eastbourne BN21 2UD, UK

**Keywords:** CAR-T cell therapy, solid tumours, biomaterials, tumour microenvironment, drug delivery systems, immunotherapy

## Abstract

Background/Objectives: Chimeric antigen receptor (CAR)-T cell therapy has achieved substantial clinical success in haematological malignancies but has shown limited efficacy against solid tumours. Key barriers include inadequate tumour trafficking, immunosuppressive tumour microenvironments, poor selectivity and heterogeneity of antigen expression, and challenges related to safety and manufacturing. Biomaterial-based technologies have emerged as a potential strategy to address many of these limitations. This review aims to critically evaluate biomaterial approaches designed to enhance CAR-T cell therapy of solid tumours and assess their translational potential. Methods: A narrative review of recent pre-clinical translational studies was conducted, focussing on biomaterial platforms developed to improve CAR-T cell delivery, persistence, functionality, safety control, and manufacturing efficiency in solid-tumour settings. Approaches were analysed according to their mechanisms of action, therapeutic benefits, and stage of translational readiness. Results: Biomaterial strategies, including nanoparticles, injectable and implantable hydrogels, scaffolds, and hybrid delivery systems, have improved CAR-T infiltration, survival, and therapeutic efficacy in several solid-tumour models. Localised delivery of cytokines and other immunomodulatory cues enabled improved spatio-temporal control of CAR-T activation, reducing systemic toxicity, and increasing persistence. Additional applications include amplified ex vivo CAR-T expansion and support for non-viral or in vivo CAR-T generation. However, increased material complexity was frequently associated with challenges in scalability, regulatory approval, and long-term safety. Conclusions: Biomaterial-enabled approaches offer a versatile toolkit to address key biological and translational barriers limiting CAR-T cell therapy of solid tumours. Strategies based on clinically familiar materials and simplified designs appear most suitable for near-term clinical translation, emphasising the need to balance engineering innovation with safety, scalability, and integration into existing clinical workflows.

## Simple Summary

Chimeric antigen receptor (CAR)-T cell therapy has been highly successful in treating certain blood cancers, but has shown limited benefit against solid tumours. This is largely due to difficulties in target selection, delivery of CAR-T cells to tumour sites, immunosuppressive tumour microenvironments, and manufacturing challenges. Biomaterials, such as nanoparticles, hydrogels, and implantable scaffolds, offer new ways to support and control CAR-T cells by improving their manufacture, localisation, activity, and persistence within solid tumours. In this review, we summarise biomaterial-based strategies designed to potentiate CAR-T cell therapy of solid tumours and discuss their potential advantages and limitations. We also consider the practical challenges that must be addressed before these approaches can be translated into clinical practice. Understanding how biomaterials can be integrated into CAR-T cell therapies may help accelerate the development of more effective treatments for patients with solid cancers.

## 1. Introduction

Chimeric antigen receptor T (CAR-T) cell therapy has transformed the management of several haematologic malignancies [1]. By contrast, efficacy against solid tumours has been limited, and there are currently no FDA-approved CAR-T products for this disease indication. This disparity is the result of multiple factors [2]. First, abnormal vasculature and dense extracellular matrix (ECM) restrict CAR-T persistence and tissue penetration [3]. Second, tumour microenvironment (TME) stressors, including hypoxia, acidosis, nutrient deprivation, and suppressive cellular and checkpoint networks hinder intra-tumoural CAR-T cell expansion and function [3]. Third, complex, costly manufacturing processes and severe side effects such as Cytokine Release Syndrome (CRS) and on-target/off-tumour effects further constrain effective clinical deployment.

Biomaterials can intervene at several of these bottlenecks by carrying cells and/or cargo, creating locally supportive niches, remodelling tissue, controlling activation and enabling in vivo monitoring. This review critically evaluates nanoparticles, injectable depots, implantable scaffolds and hybrid systems developed to improve CAR-T cell therapy of solid tumours. We compare their mechanisms, strengths, limitations and translational readiness.

## 2. Disrupting Physical Barriers and Enhancing Infiltration

**Figure 1:**
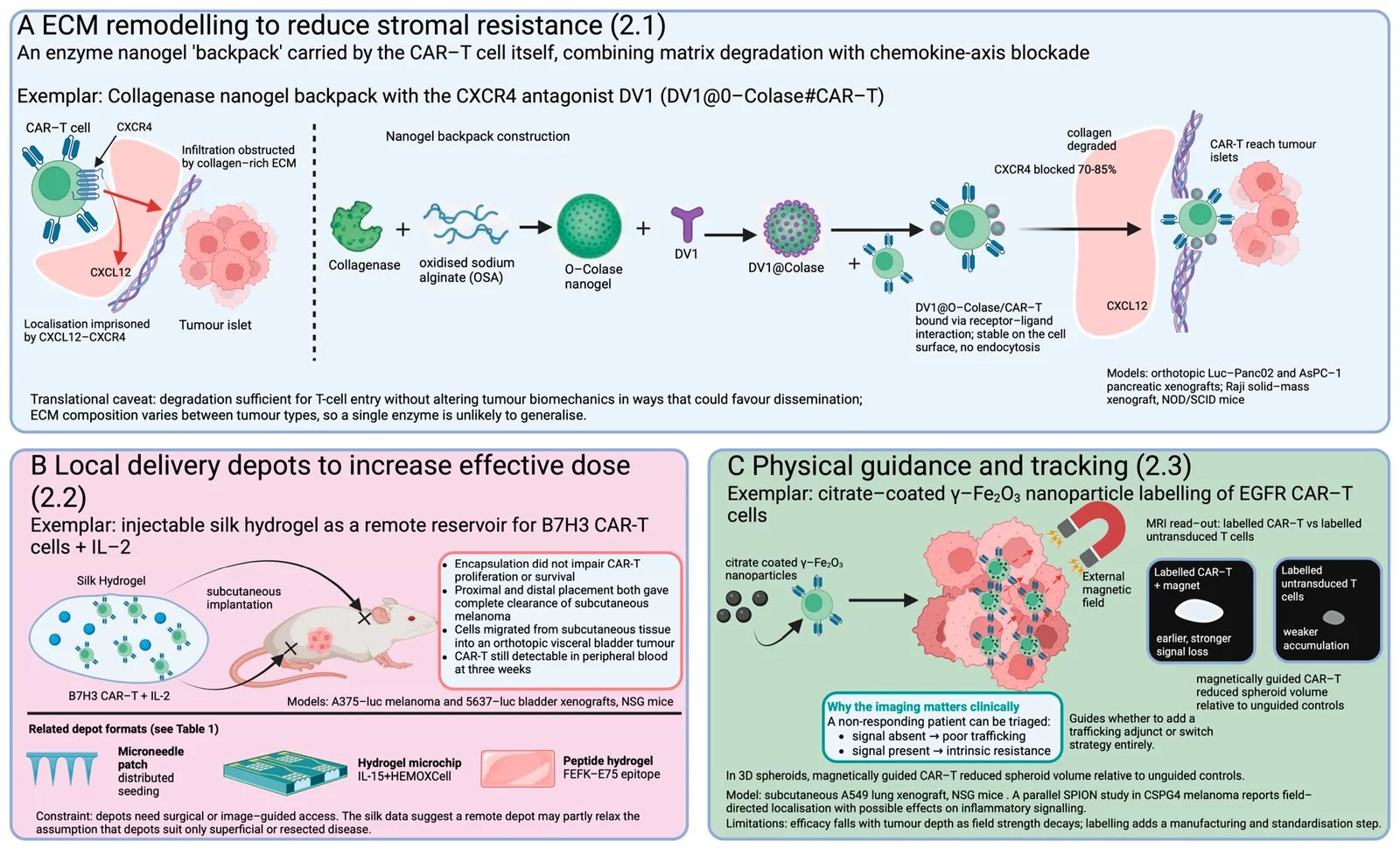
Representative biomaterial strategies that improve CAR-T cell access to solid tumours. (**A**) Cell-bound collagenase nanogels degrade ECM while the DV1 peptide blocks CXCL12-CXCR4 retention [4]. (**B**) Local depots including injectable silk and peptide hydrogels, hydrogel-microchip systems and porous microneedle patches concentrate CAR-T cells and supportive cues at or near tumour sites [6,7,8,9] (**C**) Iron oxide nanoparticle labelling enables magnetic field-assisted localisation and magnetic resonance imaging (MRI) of cell trafficking [10,11]. Created with BioRender.com.

Biomaterial strategies set out to address physical barriers within solid tumours through three complementary approaches: A. local ECM remodelling, B. spatially concentrated delivery using depots, and C. physical guidance and tracking to improve CAR-T homing. Representative biomaterial strategies are summarised in Table 1, and the principal mechanisms are illustrated in Figure 1.

### 2.1. Extracellular Matrix Remodelling to Reduce Stromal Resistance

One important strategy entails the delivery of matrix-degrading activity precisely where CAR-T cells need to penetrate. Illustrating this, “backpack” nanogels conjugated to CAR-T cell surfaces were designed to co-localise enzymatic activity with the effector cell itself [4]. In pancreatic tumour models, collagenase-crosslinked nanogels functionalised with the CXCR4 antagonist peptide DV1 combined stromal degradation with chemokine-axis disruption, increasing intra-tumoural CAR-T accumulation [4]. Similarly, hyaluronidase-loaded nanogels were hitchhiked onto CAR-T cells, enabling the degradation of hyaluronic acid-rich ECM and improved tumour penetration relative to free enzyme [5].

Although the attainment of targeted enzymatic remodelling is compelling, there is a delicate translational balance. Ideally, sufficient ECM degradation must be achieved to allow optimal T cell penetration without excess matrix damage to surrounding healthy tissues and altered tumour biomechanics, which could facilitate metastasis. Also, heterogeneity in ECM composition between tumour types may mean that a single enzymatic technique may not be generalisable. This suggests that either tumour-tailored enzymes or multi-enzyme approaches are needed, both of which increase manufacturing and regulatory complexity.

### 2.2. Local Depot Delivery to Increase the Effective Cell Dose at the Tumour Site

Injectable and implantable depots can concentrate CAR-T cells and supportive signals at a disease site, sustaining local release. Exemplifying this, hydrogel-encapsulated microchips were used to co-deliver interleukin (IL)-15 and an oxygen carrier to the TME [6]. Hydrogels can also be designed using self-assembling peptides to encompass epitopes that promote CAR-T cell proliferation and complementary vaccine delivery, triggering endogenous anti-tumour immune responses [7]. Similarly, subcutaneous silk hydrogels supported CAR-T cell persistence and enabled their migration to tumour sites [8].

Although closely related, these formats address different clinical problems. A remote subcutaneous silk depot may be implanted without entering a visceral tumour and can support cell migration to that site [8]. This approach presents a potential therapeutic opportunity for less accessible disease sites. Peptide hydrogels are injectable and molecularly programmable but require reproducible self-assembly and cargo presentation [7]. Microchip-hydrogel hybrids offer the strongest opportunity for multi-cargo support, including oxygenation, but add device manufacture and implantation complexity [6].

Porous microneedle patches instead enable the distributed seeding of CAR-T cells across superficial or surgically exposed tissue, improving their spatial dispersion relative to a bolus injection [9]. Comparative studies are required to evaluate these carriers head-to-head in an identical tumour model, with matched CAR specificity, cell dose and sampling schedule. Table 2 presents an indirect comparison and identifies the clinical setting for which each format appears best suited.

### 2.3. Physical Guidance and Tracking to Bypass Chemokine Limitations

A third approach uses physical guidance rather than tumour-derived cues to enhance tumour localisation of CAR-T cells. Labelling of CAR-T cells with iron oxide nanoparticle enables magnetic resonance imaging (MRI)-based tracking and can also permit magnetically-enhanced delivery by applying external fields over the tumour region [10]. A further advantage is reduced cytokine release by labelled CAR-T cells, potentially increasing safety [11]. Limitations include reduced efficacy with depth and the need for added labelling and standardisation steps.

Overall, biomaterial approaches to physical barriers offer a coherent engineering toolkit. Enzymatic remodelling targets stromal exclusion; depots address ineffective systemic exposure, and physical guidance improves homing while enabling monitoring. The central translational challenge is to identify platforms that deliver meaningful infiltration gains while remaining clinically deployable, scalable, and safe across heterogeneous solid tumours.

## 3. Reprogramming the TME and Enhancing Survival

**Figure 2:**
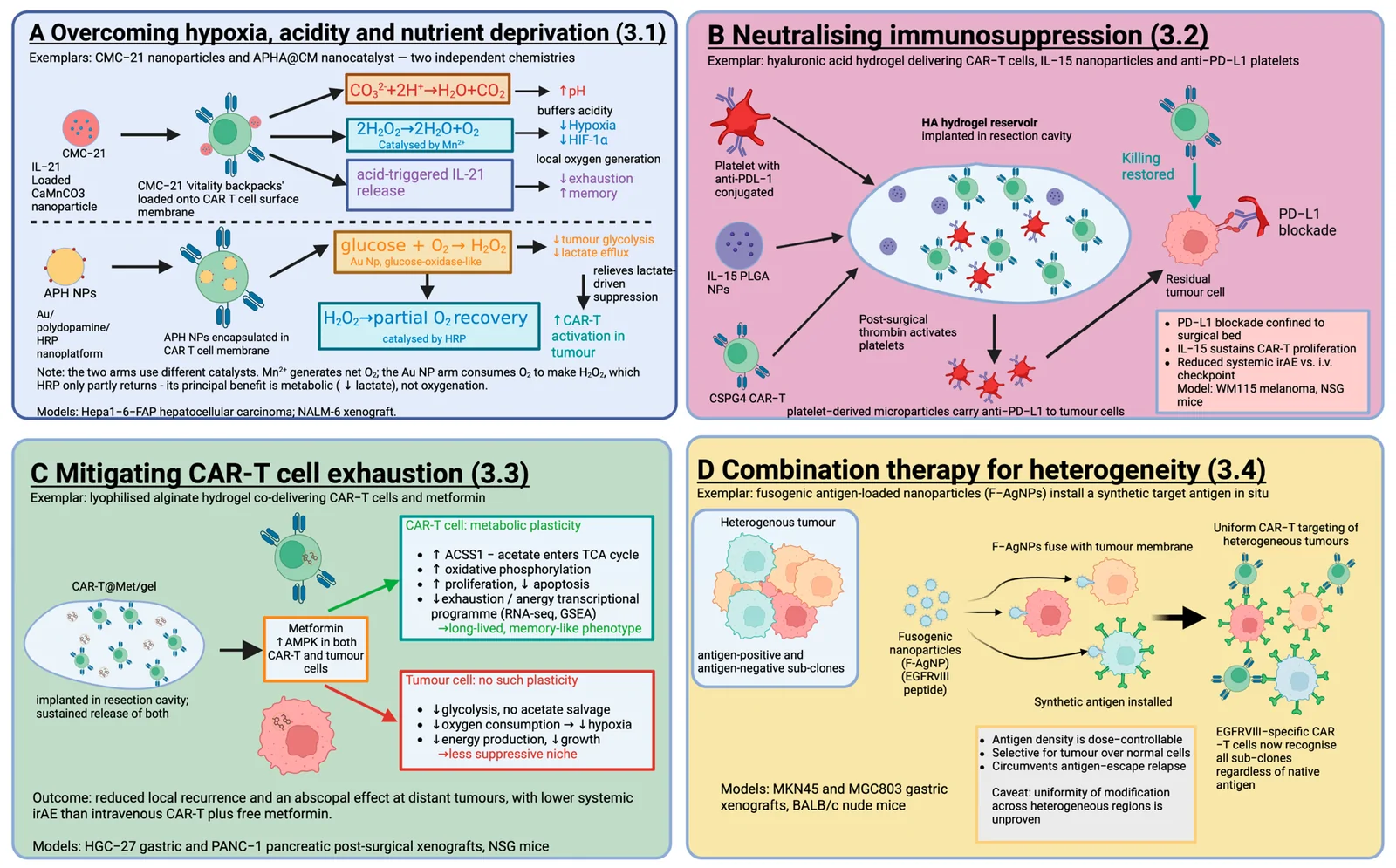
Representative biomaterials that reprogramme the TME. (**A**) Calcium manganese carbonate/IL-21 nanoparticles [13] and gold/polydopamine (Au/PDA) nanocatalysts [14] alleviate hypoxia, acidity or lactate-driven immunosuppression. (**B**) A surgically implanted hydrogel co-delivers CAR-T cells, IL-15 and platelets conjugated with anti-programmed death-ligand 1 (anti-PD-L1) [16]. (**C**) A metformin-containing alginate scaffold promotes a memory-like CAR-T phenotype, while suppressing tumour glycolysis [20]. (**D**) Fusogenic antigen-loaded nanoparticles (F-AgNPs) install a shared EGFRvIII antigenic peptide on heterogeneous tumour cells [22]. irAE, immune-related adverse events; GSEA, gene set enrichment analysis. Created with BioRender.com.

The intrinsic hostility of the solid TME is a central obstacle to CAR-T cell persistence and function [3]. Reprogramming the TME and enhancing CAR-T cell survival focuses on using biomaterials to overcome physiochemical and immunological barriers, providing localised support for CAR-T cells and converting the TME into a more favourable, stimulatory niche [3]. Key biomaterial techniques are outlined in Table 3, and the principal mechanisms are illustrated in Figure 2.

### 3.1. Overcoming Hypoxia, Acidity, and Nutrient Deprivation

The solid TME is typically acidic, hypoxic, and nutrient-deprived, compromising CAR-T cell function [24]. Biomaterials have been developed to modulate the local environment by restoring vascular function or chemically rebalancing the microenvironment. One strategy uses a poly(ethylene glycol) dimethacrylate(PEGDMA)/FeCl_2_ hydrogel for sustained release of tetramethylpyrazine. This activates vascular endothelial growth factor and the endothelial nitric oxide synthase/nitric oxide signalling to alleviate hypoxia, thereby enhancing CAR-T infiltration and persistence [12]. However, prolonged pro-angiogenic signalling could also promote tumour growth, metastasis or haemorrhage. Long-term safety and feasibility of repeated intra-tumoural delivery remains unclear.

Nanoparticles also offer the prospect of achieving dynamic TME reprogramming. Exemplifying this, calcium manganese carbonate (CMC-21) nanoparticles encapsulated with IL-21 were backpacked onto the cell surface of CAR-T cells [13]. These biodegradable particles catalyse the conversion of H_2_O_2_ into O_2_, thereby neutralising acidity and mitigating hypoxia, while also suppressing Hypoxia-Inducible Factor 1α expression. These changes favoured a shift in tumour-associated macrophages (TAMs) from an M2-like (CD206^+^) to an M1-like (CD86^+^) phenotype. A separate near-infrared (NIR) II fluorescent nanocatalyst reduced lactate and sensitised tumours to CAR-T killing after ultrasound and NIR illumination [14]. Both strategies require careful control of catalytic activity, stability and dose because therapeutic and off-target effects may share a narrow window.

The injectable i-G/MC hydrogel-microchip system also integrates oxygen carriers (HEMOXCell) and IL-15 to directly oxygenate the TME and enhance immune persistence [6], although its complexity may hinder translation to clinical settings.

### 3.2. Immune Stimulation Within the TME

Within the TME, CAR-T cells are inhibited by immune checkpoints and suppressive regulatory T cells (Tregs) and myeloid cells [3]. Biomaterials can localise countermeasures to disarm these inhibitory factors, while minimising systemic exposure. One commonly used example involved the use of nanogels that carry an IL-15 superagonist complex. These were backpacked onto CAR-T cells and underwent selective cargo delivery upon T cell activation, owing to an increase in cell surface reduction potential [15] or in response to tumour-associated hypoxia [25]. Another multifaceted approach entails the use of a hyaluronic acid hydrogel to deliver CAR-T cells, IL-15 nanoparticles, and anti-programmed death-ligand (PD-L1)-conjugated platelets post-surgery [16]. By this means, local inflammation was leveraged to release checkpoint inhibition and support CAR-T cell proliferation and persistence in the surgical resection bed. Intriguingly, abscopal control of a secondary tumour site was also achieved using this system.

Alternatively, tumour-localised inhibition of immunosuppressive metabolites such as adenosine may be achieved using a related approach. In one such example, CAR-T cells were loaded with crosslinked multi-lamellar liposomes containing the A2A adenosine receptor inhibitor, SCH-58261 [17]. Similarly, internalising RGD peptide (iRGD)-modified lipid nanoparticles (LNPs) were used to deliver a combination of phosphoinositide 3-kinase inhibitors and invariant natural killer T cell (iNKT) agonists. As a result, the TME was reprogrammed to reduce Tregs and immunosuppressive myeloid cells while stimulating immune effector cells such as iNKT cells and CD8^+^ T cells [18].

Extracellular vesicles (EVs) offer a biomimetic alternative to synthetic nanocarriers. These have been engineered to express CAR antigen, enabling them to stimulate expansion and anti-tumour activity of CAR-T cells both in vitro and in vivo [26,27]. CAR-T cells themselves also secrete EVs and maintain the ability to recognise and kill target cells which express cognate target antigen [28]. CAR-T cell derived EVs have also been engineered to co-express heparanase, enhancing their ability to penetrate ECM and achieve tumour cell killing [29]. It should be noted, however, that tumour-derived EVs may inhibit CAR-T cell function since they can express immunosuppressive molecules such as PD-L1 and are targeted to CAR-T cells via expression of cognate antigen [30].

Another approach to enhance CAR-T cell expansion and activation within the TME entails the delivery of an injectable scaffold that replicates tertiary lymphoid structures at that location [31]. When loaded with CAR-T cells, B cells and cytokines (IL-15 + IL-21), superior tumour control with abscopal tumour regression was noted using this strategy.

Importantly, some platforms combine immunomodulation with broader physical and thermal remodelling of the inhibitory niche. Illustrating this, a hydrogel system was designed to generate gold nanoparticles upon microwave ablation, thereby disabling CAR-T cell immune escape by reducing ECM viscosity and converting the TME from immunologically cold to hot [19]. However, such thermal approaches require precise control to minimise collateral tissue injury and ensure reproducibility.

### 3.3. Mitigating CAR-T Cell Exhaustion

CAR-T cells in solid tumours often experience functional exhaustion due to chronic antigen exposure and lack of supportive signals [3]. Biomaterials can help to address this by sustaining stimulatory cues and reducing stress-induced apoptosis. An elegant example entails the use of lyophilised alginate hydrogel scaffolds to deliver metformin. This approach boosted oxidative metabolism by CAR-T cells, thereby reducing apoptotic rates significantly in tumour resection models [20]. Metformin also inhibited anaerobic glycolysis by tumour cells, although reliance of this technology on surgical access limits broad applicability.

### 3.4. Combination Therapy for Heterogeneity

Antigenic heterogeneity and antigen-negative escape variants remain significant barriers to CAR-T efficacy against solid tumours [2]. Biomaterials can address this by integrating CAR-T killing with broader immune activation or by expanding/reshaping targetable antigen landscapes. Epitope spreading is emerging as a key mechanism underlying successful CAR-T cell immunotherapy of solid tumours [32].

Implantable alginate scaffolds co-delivering CAR-T cells and a STING (Stimulator of Interferon Genes) agonist (cyclic diGMP) link local tumour lysis with antigen-presenting cell activation, promoting endogenous responses against heterogeneous epitopes and limiting antigen-escape relapse [21]. Alternatively, fusogenic antigen-loaded nanoparticles (F-AgNPs) may be designed to decorate tumour membranes with exogenous antigens, redirecting CAR-T recognition independent of native antigen expression [22]. However, uniformity of modification across heterogeneous tumour regions remains uncertain.

Finally, photothermal therapy (PTT) delivered via nanomaterials with optical absorbing properties can remodel the TME by dilating blood vessels and releasing tumour-associated antigens, enhancing CAR-T infiltration [23]. However, the need for precise thermal dosing and the variability in individual patient responses may limit its reproducibility in clinical settings.

Biomaterial strategies for TME can therefore address metabolic stress, immune suppression and antigen heterogeneity through complementary mechanisms. Nevertheless, the evidence is dominated by small pre-clinical studies that employ different models, CARs, cell doses and experimental endpoints. Comparative efficacy, durability and safety cannot be inferred reliably across platforms without more harmonised and standardised study designs.

## 4. Enabling Translation–Manufacturing, Control, and Monitoring of CAR-T Cells

**Figure 3:**
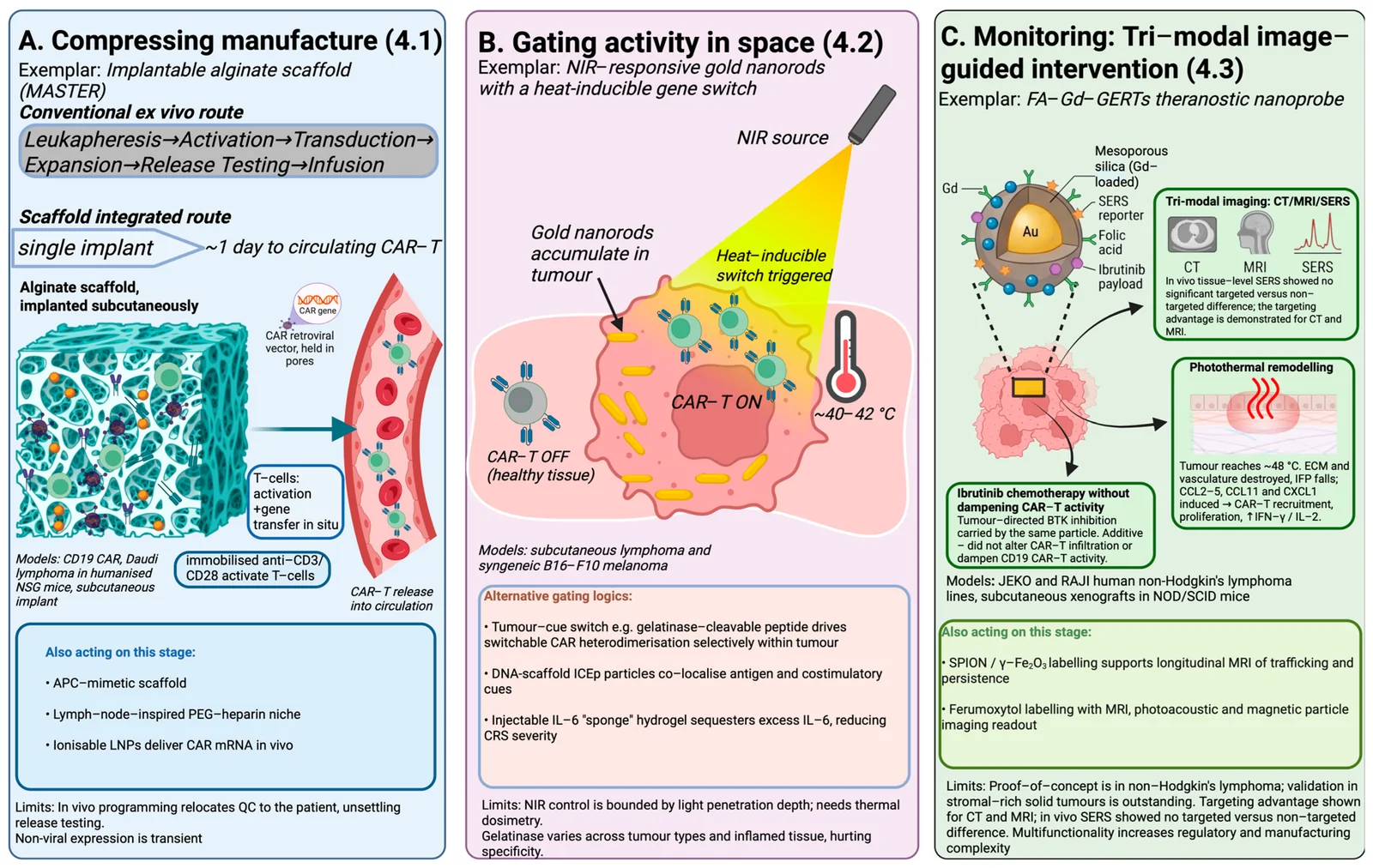
Representative biomaterial strategies enabling clinical translation. (**A**) Scaffolds, hydrogels, nanoinjection and lipid nanoparticles can improve or bypass the need for ex vivo manufacture [35,36,37,38,39]. (**B**) Systems that spatially gate CAR-T activation activity [40,41,42] or sequester derived IL-6, reducing CRS [43]. (**C**) Tri-modal imaging-guided intervention. A folate (FA)-targeted gap-enhanced Raman tag bearing an ibrutinib payload comprises a gold core within a gadolinium (Gd)-loaded mesoporous silica shell. This arrangement supports CT, MRI and surface-enhanced Raman scattering (SERS) contrast. NIR irradiation raises tumour temperature, destroying ECM, lowering interstitial fluid pressure (IFP) and inducing intra-tumoural chemokines [45]. Superparamagnetic iron oxide nanoparticle (SPION) labelling supports longitudinal MRI to monitor trafficking and persistence. QC, quality control; GMP, good manufacturing practice. Created with BioRender.com.

The translation of CAR-T cell therapy to solid tumours is also constrained by practical barriers, including complex, lengthy and expensive manufacture [33], and challenges imposed by effective monitoring of infused cells in vivo [34]. Biomaterial platforms can contribute to solving these obstacles across the CAR-T “lifecycle” by streamlining and standardising production, enabling spatio-temporally controlled activation to improve safety, and integrating diagnostic materials for real-time surveillance of therapeutic cells. Primary biomaterial studies enabling CAR-T manufacturing, safety control, and in vivo monitoring are summarised in Table 4, and the points of biomaterial intervention across the CAR-T lifecycle are illustrated in Figure 3.

### 4.1. Enhancing Manufacturing Precision and Cost-Effectiveness

Conventional CAR-T manufacture is labour-intensive, infrastructure-dependent, and typically requires weeks. Biomaterials can improve the manufacturing process by providing improved microenvironments for T cell activation, transduction, and expansion, thereby reducing batch-to-batch variability.

With respect to ex vivo manufacture, artificial antigen-presenting cell (APC)-mimetic scaffolds can out-perform paramagnetic bead-based stimulation by co-presenting activating, co-stimulatory and cytokine signals [35]. Similarly, lymph-node-inspired hydrogels can tune spatial organisation, porosity, stiffness and local factor retention to improve CAR expression and proliferation [36]. Alternatively, low-voltage nanoinjection provides non-viral gene transfer with maintained cellular viability and function, albeit with transient gene expression [39].

Implantable biomaterial systems have also been designed to enable in situ CAR-T generation. The MASTER (multifunctional alginate scaffold for T cell engineering and release) platform is a biocompatible and biodegradable scaffold designed to integrate activation, gene transfer, cytokine support and cell release, reducing processing time to approximately one day and producing CAR-T cells that subsequently enter systemic circulation [37]. Such decentralised approaches may particularly benefit solid-tumour programmes, which frequently incorporate additional design features to address trafficking, immunosuppression, and antigen heterogeneity [3,46,47]. However, in vivo programming relocates manufacturing and quality control to the patient, and therefore introduces broader translational and regulatory challenges surrounding the control of gene-delivery, product consistency, and batch release testing. Similar considerations apply to the rapidly emerging field of in vivo CAR-T cell immunotherapy, a large component of which employs targeted non-viral lipid nanoparticles [48] that deliver activating, co-stimulatory and sometime cytokine-supportive signals [49]. Non-viral in vivo CAR-T cell immunotherapy has recently been extensively reviewed [50].

### 4.2. Improving Therapeutic Precision and Safety Control

Systemic CAR-T activity can cause severe adverse effects, most notably CRS and on-target/off-tumour toxicity. These can be particularly problematic in patients with solid tumours where truly tumour-specific antigens are uncommon [51,52]. To mitigate this, biomaterials can be used to enable more precise spatio-temporal control by coupling CAR-T activation to external stimuli and/or tumour-associated cues.

Photothermal nanomaterials provide an externally addressable “on-switch” for engineered T cells. In one strategy, NIR (near-infrared)-responsive materials, such as gold nanorods, passively accumulate in tumours following systemic delivery. These generate mild hyperthermia under NIR irradiation, thereby activating heat-inducible programmes in CAR-T cells within a narrow temperature window (approximately 40–42 °C) [40]. This biomaterial-actuated control can restrict CAR-T cell activation to treated sites, improving precision and reducing systemic exposure, although feasibility may be limited by light penetration depth and the need for reliable, patient-specific thermal dosimetry [40,53,54]. Complementary tumour-cue responsive systems, such as gelatinase-responsive “nano-switch” designs, aim to localise CAR activation to protease-rich TMEs [41]. This is achieved using a switchable CAR whereby an intact heterodimeric signalling domain is assembled and functionalised by a gelatinase-generated peptide. While conceptually attractive, heterogeneity of enzyme expression across tumour types and inflammatory tissues may affect specificity and reproducibility.

An alternative approach to achieve targeted CAR-T cell activation entails the use of DNA-scaffold immune cell-engaging particles (ICEp). These biodegradable particles extend local control by enabling tunable presentation of antigens and co-stimulatory cues, supporting logic-gated activation designs that may reduce unintended systemic activation [42]. However, as platform complexity increases, translational barriers related to multi-component manufacturing, stability, and quality control also arise, requiring careful balancing of sophistication against scalability.

Biomaterials can also mitigate toxicity directly. Because IL-6 is central to CRS pathophysiology, an injectable IL-6-adsorbing hydrogel (“IL-6 sponge”) has been developed to sequester excess IL-6 and thereby reduce CRS severity [43]. As an adjunct strategy, its clinical value will depend on predictable adsorption capacity and ensuring that cytokine sequestration does not compromise anti-tumour efficacy or distort inflammatory monitoring during treatment escalation.

### 4.3. Real-Time Monitoring and Theranostics

In solid tumours, failure of CAR-T cell treatment frequently reflects inadequate trafficking and persistence, making the assessment of bio-distribution invaluable. CAR-T cells can be labelled with biodegradable SPIONs (superparamagnetic iron oxide nanoparticles) or γ-Fe_2_O_3_ nanoparticles through non-specific endocytosis without materially impairing viability or cytolytic function [10,43]. Magnetic resonance imaging can then be used to assess tumour access and help to distinguish delivery failure from intrinsic resistance [44]. However, signal dilution during cell division, label persistence after cell death and reduced magnetic guidance at depth complicate interpretation and deployment.

Finally, theranostic nanoprobes integrate imaging with therapeutic application. Multifunctional nanoparticle systems enabling multimodal imaging (computed tomography/MRI) alongside adjunct modalities, such as photothermal effects, have been used to combine diagnostic guidance with microenvironmental modulation to support CAR-T activity in pre-clinical lymphoma models [45]. This illustrates a proof-of-concept that would require validation in stromal-rich solid tumours. However, multifunctionality may increase regulatory and manufacturing complexity, reinforcing the broader translational imperative to simplify platforms without compromising efficacy.

## 5. Clinical Translation of Biomaterial-Based CAR-T Cell Therapies

The previous sections outline a wide range of biomaterial platforms that address distinct barriers to effective CAR-T cell therapy of solid tumours, spanning physical infiltration, TME reprogramming, and manufacturing/safety control. The transition to clinical application requires further consideration of how these platforms intersect with delivery logistics, manufacturing practicality, regulatory classification, and long-term safety. Here, we consider these issues and identify priorities most likely to support near-term clinical entry. Table 5 summarises representative biomaterial platforms according to their principal mechanisms of action, advantages, limitations, and clinical readiness.

### 5.1. Platform Selection: Local Versus Systemic and Injectable Versus Scaffold-Based Delivery

Biomaterial platforms to support CAR-T cell delivery broadly fall along two intersecting axes: local versus systemic administration, and injectable versus scaffold-based formats. Local strategies, including peptide and silk hydrogels, hydrogel-microchip depots, and microneedle patches, concentrate CAR-T cells and supportive cues at or near the tumour site, reducing reliance on systemic trafficking and limiting off-target exposure [6,7,8,9]. Injectable hydrogels offer a minimally invasive approach that avoids surgical placement and is compatible with image-guided or peri-operative administration, but generally provides less control over cargo release and spatial organisation than pre-fabricated scaffolds. By contrast, scaffold-based systems such as the implantable MASTER alginate platform permit greater multi-cargo integration and can combine activation, gene transfer and release within a single device [37]. However, these generally require surgical or interventional placement and more complex device manufacture.

Nanoparticles and their derivatives are better suited to intravenous delivery and repeat dosing. They are more readily compatible with disseminated or inaccessible disease, but must overcome bio-distribution and targeting challenges (e.g., exposure to lungs, liver and spleen) to achieve adequate tumour exposure [4,5,18,38].

In conclusion, platform selection should be guided by tumour accessibility, disease distribution, and the practical feasibility of incorporation within existing surgical and interventional radiology workflows. Local systems are most suited to application to superficial lesions, surgical resection beds or disease sites that are amenable to image-guided access. On the other hand, systemically delivered nanoparticles are best suited to multi-focal disease provided that selectivity and pharmacokinetic challenges can be addressed.

### 5.2. GMP Manufacturing, Reproducibility, Scalability, and Cost

Manufacturing complexity is a major determinant of translational feasibility. Conventional autologous CAR-T manufacture is complex and labour-intensive, with approved products costing up to $500,000 per dose in the United States [55]. Biomaterial platforms that integrate activation, transduction and expansion within a single device, such as artificial APC-mimetics and the MASTER scaffold, can reduce processing time, standardise activation conditions, and lower batch-to-batch variability relative to bead-based methods [35,37]. Lipid nanoparticles offer a further manufacturing advantage by enabling non-viral gene transfer that leverages existing mRNA vaccine production infrastructure, avoiding the cost and lead time associated with viral vector manufacture.

Increasing platform sophistication frequently compromises reproducibility. Complex, multi-step synthesis-based approaches are often difficult to standardise and scale to good manufacturing practice (GMP) requirements. Closed, automatable processing, defined release-testing criteria, and consistent performance across heterogeneous donor material are essential pre-requisites for clinical-grade production. Platforms built using clinically familiar, already-GMP-manufactured materials, such as alginate and lipid nanoparticles, are therefore best positioned for near-term scale-up.

### 5.3. Regulatory and Long-Term Biosafety Considerations

Standardised regulatory guidance for the long-term biocompatibility, bio-distribution, immunogenicity, genotoxicity and metabolic clearance of many nano- and micro-scale materials remains under-developed [56]. This is a particular concern for platforms involving persistent or non-biodegradable components, such as metallic nanoparticles and photothermal agents, where long-term retention, immunogenicity, and clearance pathways are incompletely characterised [40,44,45]. Chronic or repeated manipulation of the TME through sustained enzymatic remodelling or vascular reprogramming raises theoretical concerns about facilitating metastatic spread or off-target tissue damage that require longer-term pre-clinical evaluation.

Platforms that rely on in situ or in vivo generation of CAR-T cells, including implantable manufacturing scaffolds [37] and in vivo lipid nanoparticles [48,49], additionally shift quality control and dose determination from a centralised manufacturing facility to the point of care, introducing new questions around the control of gene-delivery, inter-patient product consistency, and applicability of conventional release testing. Early engagement with appropriate regulatory authorities to guide development pathways is strongly encouraged in light of these challenges.

### 5.4. Clinical Feasibility and Development Priorities

Near-term clinical entry is best supported by platforms that use biomaterials with an established safety record in humans, simpler architectures, and workflows compatible with existing care pathways (e.g., peri-operative placement into resection cavities, interventional radiology-guided injection) [57]. In terms of manufacturing, ionisable lipid nanoparticles are frontrunners, providing a safer, non-viral method for both ex vivo and in vivo T cell engineering [58], leveraging in part the success of recent mRNA vaccine platforms. Alginate-based hydrogels and scaffolds also demonstrate high potential due to their FDA-approved status [57] and ability to serve as localised “factories” that control tumour growth while minimising systemic toxicities like CRS [37].

## 6. Conclusions and Future Perspectives

**Figure 4:**
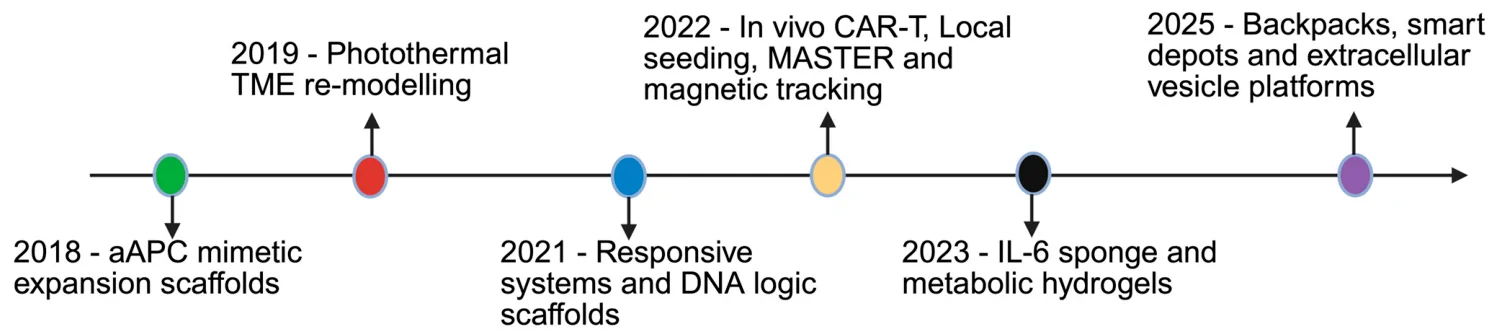
Selected milestones in the evolution of biomaterial-assisted CAR-T cell therapy. The timeline is a synthesis of representative studies discussed in this review. Dates denote publication of early proof-of-concept studies [6,7,9,10,11,12,13,18,23,29,30,37,39,42,44,45,46,49]. Please see text for details.

Biomaterial techniques are playing increasingly important role in efforts to realise durable CAR-T efficacy in patients with solid tumours as they help provide control over where, when, and how therapeutic signals and cells are delivered. Recent evolution of the field is summarised in Figure 4.

Strategies reviewed include biomaterial techniques that improve tumour access by remodelling stromal barriers, concentrate effective doses at disease sites, and support persistence by countering metabolic stress and local immunosuppression. Biomaterial techniques can also improve manufacture, introduce control “switches” to reduce systemic toxicity, and support non-invasive monitoring of trafficking and persistence.

It should be noted nonetheless that significant barriers to clinical implementation remain. Long-term safety continues to be incompletely defined for many complex nano/micro-systems, including bio-distribution, persistence/clearance, immunogenicity, and risks associated with chronic microenvironmental manipulation (vascular and matrix remodelling) [57]. Scalability is a critical issue; complex, multi-step synthesis often lacks the batch-to-batch consistency and reproducibility required for GMP certification [59]. Furthermore, the regulatory landscape is still maturing. Hybrid products combining biological cells with synthetic materials are categorised as complex “advanced therapies” that lack standardised safety guidelines for long-term biocompatibility and metabolic clearance [56]. Finally, clinical deployment can be constrained by delivery route (reliance on intra-tumoural placement or surgical access) and by heterogeneity across lesions, patients, and tumour compartments, which can undermine reproducibility even when the underlying mechanism of action is sound [60].

Several emerging directions are likely to shape the next phase of development of the field. Artificial intelligence and machine learning are increasingly being applied to guide “smart” and patient-specific biomaterial design, accelerating the screening of candidate formulations with tailored degradation, release and immunomodulatory properties [61]. Also emerging is a shift towards personalised biomaterials, in which scaffold, hydrogel or particle properties are tuned to individual patient or tumour characteristics. Smart, stimuli-responsive systems that couple CAR-T activation to external triggers or tumour-derived cues are also expected to mature beyond current proof-of-concept, offering finer spatio-temporal control. Multifunctional theranostic platforms that combine imaging with therapeutic delivery are likely to expand similarly, providing real-time feedback on bio-distribution and treatment response, although their added complexity will need to be balanced against regulatory and manufacturing feasibility. Another rapidly emerging strategy entails the use of cell-membrane-coated nanoparticles, which can reduce immunogenicity while maintaining tumour targeting [62]. At the manufacturing level, further progress will require simplification and standardisation of material preparation and assembly, with scalable fabrication routes to improve reproducibility and reduce batch-to-batch variance [63].

Clinical translation has begun at the platform level, heralded by the use of targeted lipid nanoparticles carrying CAR messenger RNA, which are currently undergoing Phase 1 evaluation in multiple clinical trials [58].

In conclusion, biomaterials offer a versatile toolkit to improve CAR-T trafficking, persistence, metabolic fitness, safety control and manufacture. Nanoparticles favour systemic reach and programmability; hydrogels favour injectable local control; scaffolds favour structured and prolonged support; and biomimetic or responsive systems offer emerging precision. No platform is universally superior. Translation will depend on matching the material to the anatomical and biological problem while demonstrating reproducibility, long-term safety, scalable GMP manufacture, cost-effectiveness and practical feasibility in patients. Given the substantial unmet need in solid tumours, which represent the vast majority of the global cancer burden relative to haematologic malignancies [64], continued development of biomaterial-enabled CAR-T platforms is well justified. The central challenge for the next phase of development is not only to demonstrate efficacy but also to identify designs that remain safe, manufacturable, and operationally feasible at clinical scale while retaining effectiveness across heterogeneous solid-tumour environments.

## Figures and Tables

**Table 1:** Representative strategies to disrupt physical barriers and enhance infiltration.

Biomaterial Type	Cargo/Payload	Tumour Models	Strategy/Mechanism	Ref
Cell-surface nanogel backpack	Collagenase and DV1 peptide	Pancreatic cancer models (orthotopic murine Luc-Panc02; human xenograft AsPC-1).	Collagenase promotes ECM degradation while DV1 blocks CXCR4/CXCL12 stromal retention, improving CAR-T infiltration into fibrotic tumours.	[4]
Cell-surface nanogel	Hyaluronidase	Human Raji solid-mass xenograft in NOD/SCID mice.	CAR-T “hitchhiking” delivers deep into tumour tissue to degrade hyaluronic acid, loosen ECM, and increase intra-tumoural CAR-T penetration.	[5]
Hydrogel-encapsulated microchip	IL-15 + HEMOXCell	Human ovarian adenocarcinoma SKOV-3 xenograft in highly immunodeficient NPG/Vst (NOD-*Prkdc scid Il2rgnull*) male mice.	Local hydrogel-microchip depot concentrates and sustains CAR-T delivery at the tumour site, reducing reliance on systemic homing and improving local infiltration/exposure.	[6]
Supramolecular peptide hydrogel	HER2-derived E75 epitope vaccine	Breast Cancer: Humanised NSG mice models using SKBR3 (HER2-positive) and MDA-MB231 (HER2-negative) cell lines.	Provides a local matrix that co-localises immune cues with CAR-T cells to support localisation and promote more effective tumour engagement.	[7]
Injectable silk hydrogel	B7H3-targeted CAR-T cells and IL-2	Melanoma and Bladder Cancer: Subcutaneous human melanomas (A375-luc) and orthotopic human bladder tumours (5637-luc) in NSG mice.	A distant depot enables sustained release/viability, allowing CAR-T migration into tumours and improving clearance without direct intra-tumoural injection.	[8]
Porous microneedle patch	CSPG4-targeted CAR-T cells (melanoma); B7-H3-targeted (pancreatic)	Melanoma and Pancreatic Cancer: Post-surgical resection of melanoma (WM115) and orthotopic pancreatic tumour (Panc-1) models.	Microneedles physically breach tumour tissue and deliver scattered seeding for improved spatial distribution and infiltration compared with bolus intra-tumoural injection.	[9]
Magnetic iron oxide nanoparticles	EGFR-targeted CAR-T cells	Lung Cancer: Subcutaneous human lung cancer cell-derived xenografts (A549) in NSG mice.	Enables MRI tracking and magnet-assisted tumour focussing to increase early accumulation and infiltration independent of endogenous chemotaxis.	[10]
Superparamagnetic iron oxide nanoparticles (SPIONs)	CSPG4-specific CAR-T cells	Melanoma: Human melanoma cell lines (A375M, CRMM2, UPMM3) and multicellular 3D spheroids.	Magnetic loading supports field-directed localisation and may modulate inflammatory signalling and tumour cell death pathways to improve delivery control and efficacy.	[11]

**Table 2:** Comparison of local CAR-T depot formats. No head-to-head study under matched tumour conditions is available.

Carrier	Sustained Exposure	Infiltration Evidence	Operational Difficulty	Most Plausible Clinical Fit
Injectable silk hydrogel [8]	Sustained cell viability and release reported	Migration from a subcutaneous depot to orthotopic tumours	Moderate: injection/implantation; no direct tumour access required	Deep or inaccessible lesions when systemic migration remains adequate
Supramolecular peptide hydrogel [7]	Programmable self-assembly; duration depends on peptide degradation and cargo binding	Local expansion plus vaccine-supported endogenous immunity	Moderate: injectable, but assembly and epitope quality control are critical	Accessible lesions requiring combined local CAR-T and vaccination
Hydrogel-encapsulated microchip [6]	Designed for sustained multi-cargo support, including IL-15 and oxygen	Local retention, survival and infiltration from an engineered immune niche	High: device fabrication, loading and implantation are required	Post-operative or image-guided placement
Porous microneedle patch [9]	Primarily spatially distributed delivery rather than prolonged reservoir release	Broad, shallow seeding across exposed tumour or resection bed	Moderate to high: patch placement requires superficial or surgical access	Superficial tumours and post-resection beds

**Table 3:** Representative strategies to reprogramme the TME and enhance survival.

Biomaterial Type	Cargo/Payload	Tumour Model	Strategy/Mechanism	Ref
Injectable hydrogel	Tetramethylpyrazine	Triple-negative breast cancer xenograft in the mammary fat pad of female Balb/c nude mice	Activates vascular endothelial growth factor expression to remodel vasculature, relieve hypoxia and improve CAR-T infiltration and function.	[12]
Calcium manganese carbonate nanoparticles	IL-21	Hepa1-6-FAP murine hepatocellular carcinoma in mice	Buffers tumour acidity and generates oxygen to reverse hypoxia, while polarising TAMs from M2 to M1.	[13]
Gold nanoparticles	Nanocatalyst	Humanised NALM-6-tumour-bearing NCG mouse model	Reduces glycolysis and lactate accumulation by inhibiting metabolic pathways, thereby improving the tumour metabolic environment for CAR-T cells.	[14]
Hydrogel-encapsulated microchip	IL-15 + HEMOXCell	Human ovarian adenocarcinoma SKOV-3 xenograft in highly immunodeficient NPG/Vst (NOD-Prkdcscid Il2rgnull) male mice	Establishes an immune-permissive niche by delivering CAR-T cells, IL-15, and oxygen carriers, enhancing T cell survival and downregulating Hypoxia-Inducible Factor-1α.	[6]
T cell tethered nanogels (NGs)	IL-15 superagonist complex	Subcutaneous B16F10 melanoma in C57BL/6 mice Subcutaneous U-87 MG human glioblastoma model in NSG mice	Nanogels affixed to T cells release IL-15 superagonist in a redox-dependent manner at tumour sites, selectively expanding T cells with minimal off-target effects.	[15]
Hyaluronic acid hydrogel	IL-15 + anti-PD-L1 platelets	Human WM115 melanoma xenograft implanted subcutaneously in NSG (NOD-scid IL2rg null) mice	Locally releases anti-PD-L1 antibodies and IL-15 in the surgical bed to reduce immunosuppression and enhance CAR-T proliferation post-resection.	[16]
Crosslinked multilayer liposome nanoparticle	A2a receptor antagonist	Subcutaneous SKOV3.CD19 human ovarian cancer xenograft in NSG mice	Delivers A2a receptor antagonist directly to CAR-T cells to block inhibitory adenosine signalling.	[17]
iRGD-lipid nanoparticles	PI3K inhibitors + iNKT cell agonists	Orthotopic 4T1 murine breast cancer model in BALB/cJ mice	Uses peptide-modified lipid nanoparticles to deliver immunomodulators that deplete suppressor cells and convert the TME into a stimulatory niche.	[18]
In situ gold bioreactor + microwave ablation	Thermal/ECM modulation platform	In vivo CAR-T experiments: MDA-MB-1 human breast cancer xenograft model in NSG mice Non-CAR-T mechanistic ablation studies: 4T1 murine breast tumours in Balb/c mice	Combines microwave ablation with in situ bioreactor synthesis to remodel ECM viscosity and deplete Tregs and M2 macrophages, alleviating immunosuppression.	[19]
Lyophilised alginate hydrogel	CAR-T cells + metformin	Gastric model: Human HGC-27 cells Pancreatic model: Human PANC-1 cells inoculated subcutaneously in NSG mice	Co-delivers CAR-T cells and metformin to the resection site, reprogramming them toward an activated, apoptosis-resistant phenotype for durable response.	[20]
Alginate biopolymer scaffold	CAR-T cells + STING agonist (cdGMP)	Pancreatic ductal adenocarcinoma melanoma (B16F10)	Delivers CAR-T cells and a STING agonist via an implantable scaffold to promote tumour antigen release and initiate systemic T cell responses against escape variants.	[21]
Fusogenic antigen-loaded nanoparticles	Exogenous antigens	Human gastric cancer xenografts: subcutaneous MKN45 tumours and peritoneally disseminated MGC803-luc tumours in female BALB/c nude mice	Modifies tumour membranes with exogenous antigens to create universal CAR-T targets, overcoming heterogeneity and antigen escape.	[22]
ICG-PLGA or Au nanorods	Photothermal agents	Subcutaneous human melanoma xenograft using the WM115 cell line implanted in NSG mice	Uses photothermal agents to remodel vasculature, promote antigen release, and synergise with CAR-T cell therapy for deeper tumour penetration and systemic immunity.	[23]

**Table 4:** Representative strategies to enable translation of CAR-T Cells.

Biomaterial Platform	Use	Engineering Input	Validation Setting	Key Mechanism/Outcome	Ref
Artificial antigen-presenting cell (APC) scaffold system	Manufacturing	Lipid bilayers functionalised with anti-CD3/CD28 antibodies and IL-2	Ex vivo: human primary T cells; In vivo: murine E.G7-OVA lymphoma	Mimics natural APC-T cell interactions; expanded CAR-T cells 5-fold more than conventional beads by providing a more physiologic niche.	[35]
Bio-hybrid hydrogels	Manufacturing	Intermediate stiffness (3.1 kPa); interconnected 120 μm pores; heparin-virus affinity	Ex vivo: primary human CD4^+^ T cells; anti-CD19 CAR construct	Bio-hybrid hydrogels recreate a 3D lymphoid-like niche with optimal stiffness, using heparin to enhance virus-cell binding and boost CAR-T transduction and expansion.	[36]
Multifunctional Alginate Scaffold (MASTER)	Manufacturing	Immobilised anti-CD3/CD28; encapsulated IL-2; CD19 CAR retrovirus	In vivo: Subcutaneous Daudi lymphoma in humanised NSG mice	Reduces manufacturing to a single day; avoids ex vivo differentiation to produce long-lasting memory phenotypes with superior persistence.	[37]
Ionisable Lipid Nanoparticles (LNPs)	Manufacturing	mRNA encoding CD19-targeted CAR; ionisable lipid core	In vitro: Nalm-6 ALL cells; In vivo: Leukaemic murine models	Provides a non-viral alternative for in vivo engineering, eliminating complex ex vivo manipulation and reducing genotoxicity risks.	[38]
Electroactive Nanoinjection	Manufacturing	Low-voltage electrical pulses (10–40 V); vertically aligned nanotube tips	Ex vivo: primary human T cells; anti-CD19 CAR construct	Nano-electroporation creates precise, transient pores; achieved 68% transfection efficiency with significantly higher viability than bulk methods.	[39]
Photothermal-control system (e.g., Au nanomaterials)	Safety control/Precision	Heat-activated gene switch; gold nanorods; IL-15 superagonist	In vivo: subcutaneous lymphoma and syngeneic B16-F10 melanoma models	NIR light induces mild hyperthermia (40–42 °C) via nanorods to trigger localised CAR expression only within the illuminated tumour mass.	[40]
Gelatinase-responsive Nano-Switch	Safety control/Precision	Gelatinase-responsive peptide; switchable heterodimerisation molecules	In vivo: HGC-27 gastric cancer xenograft model in NSG mice	Nanoparticles release “switch” molecules only in the TME where gelatinase activity is high; ensures precision CAR-T activation within tumours.	[41]
DNA scaffolds on particles (ICEp)	Safety control/Precision	Short synthetic DNA scaffolds; co-localised antigens and co-stimulatory cues	In vivo: B16-OVA melanoma syngeneic mouse model	Implements an AND-gate logic; systemically infused T cells only activate upon reaching the high local density of ICEp particles in tumours.	[42]
IL-6 adsorbing hydrogel	Safety control	Temperature-sensitive hydrogel; IL-6-specific neutralising antibody	In vivo: CD19-targeted CAR-T models (Nalm-6 or Raji-luc) in NSG mice	Subcutaneously injected hydrogel acts as a systemic “sponge” to absorb excess IL-6, significantly mitigating CRS severity.	[43]
γ-Fe_2_O_3_ Iron Oxide NPs (IONPs)	Monitoring/ Precision	Intracellular iron oxide label; external magnetic field guidance	In vivo: A549 human lung cancer subcutaneous xenograft in NSG mice	Enables longitudinal MRI tracking for up to 14 days and use of magnets to physically attract CAR-T cells into the tumour.	[10]
Magnetic nanoparticle labelling (Ferumoxytol)	Monitoring	Ferumoxytol iron oxide label; microfluidics-based convection labelling	In vivo: B7-H3 CAR-T cells in osteosarcoma (MG63.3) models	Non-invasive detection using MRI, Photoacoustic and magnetic particle imaging allows for real-time visualisation of cell trafficking and accumulation.	[44]
FA-Gd-GERTs nanoprobes (Theranostic NPs)	Monitoring/Safety	Gold core; Gadolinium-loaded mesoporous silica shell; folate targeting; ibrutinib	In vivo: Non-Hodgkin’s lymphoma (NHL) subcutaneous tumour models	Tri-modal imaging guides therapy; photothermal effect loosens dense tissue while ibrutinib/CAR-T provides synergistic killing.	[45]

**Table 5:** Summary of biomaterial platforms for clinical translation of CAR-T cell therapy of solid tumours.

Platform	Principal Mechanisms	Advantages	Limitations	Clinical Readiness
Nanoparticles	Deliver CAR encoding nucleic acid, drugs, enzymes, imaging agents or cytokines systemically or from the CAR-T surface	Injectable, tunable, versatile, compatible with in vivo engineering	Risk of off-target delivery, transient expression	Mainly pre-clinical. Lipid nanoparticles have the strongest clinical and manufacturing precedent [4,5,18,38]
Injectable local depots	Sustained local release/seeding of CAR-T cells and supportive agents at accessible sites	High local exposure. Minimally invasive when injectable	Requires lesion access. Limited multi-lesion coverage	Pre-clinical; plausible near-term use with clinically precedented hydrogels [6,7,8]
Implantable scaffolds/devices	In situ CAR-T production from a biodegradable scaffold	Capacity to deliver large numbers of CAR-T and supportive factors. Single-step rapid manufacture	Relocates manufacture/QC to the patient; requires interventional placement	Pre-clinical; best aligned with planned surgery [37]
Microneedle patches	Distributed local seeding of CAR-T across tissue via physical microneedles	Improved spatial coverage without high pressure delivery	Requires superficial or surgically exposed site. Medical device regulatory requirements	Pre-clinical [9]
Biomimetic carriers	Extracellular vesicles to stimulate, complement or even replace CAR-T cells	Targeted to CAR-T cells, may improve safety	Batch-to-batch variability, potency, purity and storage challenges	Pre-clinical [26,27,28,29]
Stimuli- responsive systems	External or tumour-cue-gated activation of CAR signalling to spatially restrict activity	Improved spatial and temporal precision, potential to reduce toxicity	Added multi-component complexity; trigger and tissue heterogeneity limit reproducibility	Early pre-clinical [40,41,42]
Theranostic nanoprobes	Non-invasive tracking (e.g., MRI/) ± magnetically-guided delivery	Enables bio-distribution monitoring and early response assessment	Signal dilution over time, reduced magnetic guidance at depth, added regulatory complexity for multifunctional agents	Pre-clinical proof-of-concept [10,44,45]

## Data Availability

No new data were created or analysed in this study. Data sharing is not applicable to this article.
